# Compulsory education law and intergenerational income mobility in China

**DOI:** 10.3389/fsoc.2025.1662919

**Published:** 2025-11-28

**Authors:** Qingqing Yuan, Zeyun Liu, Hewen Wu

**Affiliations:** 1 Faculty of Education, Shaanxi Normal University, Xi'an, China; 2 Business School, Beijing Normal University, Beijing, China

**Keywords:** intergenerational income mobility, compulsory education law, educational inequality, social stratification, China, occupational inequality

## Abstract

**Introduction:**

Intergenerational income mobility serves as a key indicator of equality of opportunity and social stratification. Education is widely seen as a vital pathway for enhancing social mobility. This paper examines China’s Compulsory Education Law of 1986 and analyzes its effects on intergenerational income mobility.

**Methods:**

Using data from the China Household Income Project, this study constructs matched samples of parents and adult children. Income adjustment and Heckman selection models are employed to correct measurement errors and co-residence selection bias. A difference-in-differences approach is used to identify the causal effects of the policy, supplemented by triple differences, event-study designs, and other robustness checks.

**Results:**

The study finds that the Compulsory Education Law significantly reduced intergenerational income correlation, thereby increasing income mobility. The mechanism analysis shows that the policy significantly reduced inequalities in access to junior secondary education and non-agricultural employment opportunities, thereby enhancing intergenerational income mobility. Yet, persistent inequalities in access to senior secondary education and high-status occupations, together with class-based differences in returns to education, may have partially offset these gains. The heterogeneity analysis further shows that the policy had a stronger effect on intergenerational mobility among urban households compared with rural ones, while no significant differences were observed by gender.

**Discussion:**

Expanding access to compulsory education can, to some extent, weaken the intergenerational transmission of economic advantages and promote social mobility, although its effects vary across social groups. The findings provide empirical evidence for ongoing policy debates on whether to extend the duration of compulsory education and offer broader insights into the dynamics of social mobility in developing economies.

## 1 Introduction

Intergenerational income mobility, a key measure of equality of opportunity (Becker and Tomes, 1986; Corak, 2013) and a central concern in the study of social stratification (Torche, 2015), shows how much children’s economic outcomes are unrelated to their parents’ outcomes. Persistent low intergenerational mobility indicates entrenched social stratification, where advantages and disadvantages are passed down across generations, thus reproducing social inequality and undermining economic efficiency and social cohesion (Chetty et al., 2014; Bloome et al., 2018). As global income inequality intensifies (Piketty, 2014), intergenerational income mobility remains low in many countries, with severe social stratification, except in a few high-mobility societies such as those in Scandinavia (Björklund et al., 2009; Helsø, 2021; Fan et al., 2021; Soria and Medina, 2025). Therefore, how public policies can enhance intergenerational income mobility has become an urgent issue that requires addressing (Breen, 2010; Jácome et al., 2025).

Education is considered a key mechanism for promoting intergenerational mobility (Breen, 2010; Becker et al., 2018). As a result, educational policies have long been viewed as one of the most effective public policy tools for enhancing social mobility (Heckman and Mosso, 2014; Björklund and Jäntti, 2020). Compared to higher education reforms, focusing on compulsory education policies holds unique significance: its universality and early-stage implementation enable it to shape human capital before individuals enter the labor market (Heckman and Carneiro, 2003; Holmlund, 2008; Betthäuser, 2017). However, the equalizing effect of compulsory education expansion is not guaranteed theoretically (Raftery and Hout, 1993; Lucas, 2001). International studies also indicate that the effects of compulsory education reforms are highly context-dependent: in some European countries such as Finland and Germany, it has significantly promoted social mobility (Pekkarinen et al., 2009; Betthäuser, 2017); whereas in the United Kingdom and the United States, its impact has been limited or even negligible (Rauscher, 2016; Sturgis and Buscha, 2015). Therefore, evaluations that are specific to the context are necessary, considering the institutional environments and inequality baselines.

China provides a particularly valuable case for study. Since the 1980s, China has implemented a series of major educational reforms, including the 1986 Compulsory Education Law (CEL), the 1999 expansion of higher education, and the 2006 universal free compulsory education policy. These reforms have significantly increased the nation’s educational attainment, but their long-term effects on intergenerational income mobility remain underexplored. Among these reforms, the CEL is a key policy for assessing the redistributive potential of basic education (Yang et al., 2015). Differences in the pace of policy implementation and supporting investments across regions, combined with China’s dual urban–rural structure and uneven distribution of educational resources, have led to potential redistributive effects of compulsory education, but also heterogeneous impacts on different groups. This institutional context provides an opportunity to examine the relationship between compulsory education reforms and intergenerational income mobility, as well as to test the policy’s effects across different groups. Existing studies on China have mainly focused on the impact of educational policies on intergenerational educational or occupational mobility, with a predominant focus on higher education expansion policies, leading to inconsistent conclusions (Guo et al., 2019; Liu and Wan, 2019). Moreover, causal studies on intergenerational income mobility are extremely limited, and there is a lack of systematic empirical research on the long-term effects of the 1986 CEL on income mobility.

This study uses data from the China Household Income Project (CHIP) for the years 1995, 2002, 2013, and 2018. It constructs a difference-in-differences (DID) model based on the provincial variation in the timing of policy implementation to estimate the long-term effects of the 1986 Compulsory Education Law on intergenerational income mobility. The study addresses two major econometric challenges: income measurement error and co-residence selection bias. The former is mitigated through variable adjustments and sample restrictions, while the latter is addressed using the Heckman selection model, a technique seldom applied in studies of intergenerational mobility in the income dimension. Additionally, the study investigates the impact of the CEL on educational opportunity inequality, occupational opportunity inequality, and differential returns to education, identifying the mechanisms through which the policy influenced intergenerational income mobility. Finally, the study analyzes the heterogeneity of policy effects by urban–rural household registration status and gender.

This paper makes three main contributions.

First, in the field of social stratification, this study focuses on income as the key outcome, examining how the compulsory education reform influences opportunity equality in a unified framework. It provides new causal evidence from a large developing country, revealing how foundational education policies affect long-term intergenerational income mobility. The study directly links compulsory education reform to reducing social stratification and promoting opportunity equality, complementing the existing literature that has largely focused on higher education reform or developed countries.

Second, in terms of research design, this study combines difference-in-differences estimation with the Heckman selection model to jointly address two critical issues: income measurement error and co-residence selection bias. These issues are central to intergenerational mobility research but are often overlooked in sociological analyses (Deutscher and Mazumder, 2023). This methodological approach provides a framework that can be used in future research and enhances the robustness of the estimates.

Third, the paper not only examines the effects of the Compulsory Education Law on intergenerational income mobility but also investigates the mechanisms behind its impact on social stratification, including educational and occupational opportunity inequality, as well as class differences in returns to education. The analysis incorporates intergenerational educational and occupational mobility as potential mechanisms, establishing a comprehensive framework across the dimensions of education, occupation, and income. Furthermore, the paper highlights the heterogeneity of the policy effects based on household registration status.

This research provides differentiated policy insights for extending compulsory education in China and offers references for other developing countries seeking to restructure social stratification and enhance intergenerational mobility through investments in basic education.

## 2 Literature review, theoretical framework, and hypotheses

### 2.1 Literature review

#### 2.1.1 Disciplinary scope and measures of intergenerational mobility research

Intergenerational mobility has been a central theme in sociological research and continues to attract significant attention across other disciplines, particularly economics (Torche, 2015; Björklund and Jäntti, 2020). This body of work spans multiple dimensions, including family dynamics, social class, educational opportunities, labor market institutions, and economic development (Blau and Duncan, 1967; Featherman and Hauser, 1978). Within the disciplinary traditions, sociology tends to prioritize the analysis of occupations and social class, using socioeconomic status indicators that integrate education, occupation, and income (Sakamoto and Wang, 2020). In contrast, economics has traditionally focused on monetary indicators such as income to capture mobility (Corak, 2013; Barone et al., 2022). Both fields, however, extensively use educational indicators to assess equality of educational opportunities and their relationship with social mobility (Björklund and Jäntti, 2020). In recent years, as income inequality has intensified globally, income-based indicators have increasingly become a focal point in research on social stratification and mobility, with a growing body of sociological studies analyzing intergenerational income mobility (Bloome et al., 2018; Sakamoto and Wang, 2020; Barone et al., 2022).

While income mobility research faces challenges such as limited data availability, life-cycle bias, and measurement error, its primary advantage lies in the ability to directly link family living standards and material well-being, thereby providing a more accurate reflection of opportunity inequality. Simultaneously, improvements in data availability and advancements in econometric techniques have fueled progress in this field (Chetty et al., 2014; Emran et al., 2018; Deutscher and Mazumder, 2023). On the other hand, while occupational indicators are stable and less prone to measurement error, they are often insufficient in capturing the multidimensional aspects of income inequality and job quality (Barone et al., 2022; Haeck and Laliberté, 2025). Educational indicators, although widely used, struggle to fully capture the stratified returns to education and the mechanisms through which these returns are translated into labor market outcomes (Van De Werfhorst, 2024).

It is important to note that both education and occupation not only serve as independent variables of interest but also function as critical mechanisms of income mobility. Consequently, recent research has increasingly adopted a combined approach, focusing on income as the core dimension while integrating educational and occupational factors to provide a more comprehensive understanding of intergenerational mobility and its associated inequalities.

#### 2.1.2 Research on the impact of education on intergenerational mobility

The role of education in intergenerational mobility is a central concern in both sociology and economics. Economic theory emphasizes that education, as a key form of human capital, is central to intergenerational income mobility (Becker and Tomes, 1986; Becker et al., 2018). Based on this, research has primarily focused on the extent of education’s role in intergenerational income mobility (Gregg et al., 2017; Harding and Munk, 2020), the impact of public education spending and structure on intergenerational income mobility (Mayer and Lopoo, 2008; Biasi, 2023; Yuan et al., 2023), and the effects of educational policies (Pekkarinen et al., 2009; Guo et al., 2019). Sociology, in contrast, has proposed richer theoretical frameworks, such as the status attainment model, which emphasizes the role of education in the intergenerational transmission of social and economic status (Blau and Duncan, 1967). The cultural capital theory argues that education reproduces social inequalities through cultural capital differences (Bourdieu, 1973), while modernization theory highlights how educational expansion meets skill demands and enhances intergenerational mobility (Treiman, 1970).

At the same time, the Maximally Maintained Inequality (MMI) theory and the Effective Maintenance of Inequality (EMI) theory suggest that educational expansion does not automatically lead to greater mobility. Instead, the distribution of educational resources inherently includes mechanisms of class competition and a “quality substitution for quantity” logic. Specifically, families from higher socioeconomic backgrounds can leverage various resources and strategies during periods of educational expansion to ensure that their children maintain priority positions in school admissions and transitions. After educational universalization, they may then use selective high-quality schools and elite curricula to maintain and even strengthen their relative advantage in terms of educational quality (Raftery and Hout, 1993; Lucas, 2001).

Educational expansion policies provide an important institutional setting to test these theories. Representative reforms include the Compulsory Education Law (CEL), the minimum school-leaving age (or extension of compulsory education), and the expansion of higher education (Sturgis and Buscha, 2015; Betthäuser, 2017; Grätz, 2022; Espadafor and García-Sierra, 2025). In European and American countries, early studies have primarily relied on trend descriptions or pre- and post-reform comparisons, which made it difficult to draw causal conclusions (Sturgis and Buscha, 2015; Betthäuser, 2017). However, with the introduction of methods such as difference-in-differences, regression discontinuity, and instrumental variables, research has progressively shown that compulsory education reforms in several European countries significantly enhanced educational mobility and reduced educational inequality (Grätz, 2022). However, evidence on occupational mobility remains limited, with positive effects found only in West Germany (Betthäuser, 2017). Research on intergenerational income mobility remains relatively scarce, but existing evidence suggests that compulsory education reforms in Finland and Sweden have enhanced intergenerational income mobility (Pekkarinen et al., 2009; Holmlund, 2008).

In China, findings are heterogeneous. Some evaluations of the 1986 Compulsory Education Law suggest that it enhanced educational mobility, particularly benefiting children from families with lower parental education, occupation, or income levels (Chen et al., 2021). However, other studies report divergent results. Guo et al. (2019) find negative effects on educational mobility among rural populations, with no significant impact on urban groups, while Peng and Zhao (2021) report that the policy did not significantly alter rural educational mobility, except for upward mobility among individuals from disadvantaged backgrounds. These discrepancies highlight the importance of the urban–rural dual structure and uneven distribution of educational resources in shaping policy outcomes in China. Beyond compulsory education, the expansion of higher education has also received attention. Studies suggest it promoted educational and occupational mobility to some extent (Liu and Wan, 2019), yet it decreased income mobility, with effects primarily driven by female cohorts (Zhou and Xu, 2017).

These studies indicate that the impact of educational expansion on intergenerational mobility varies significantly across countries and social groups. Although the expansion of compulsory education is widely regarded as an important system for reducing educational inequality, its causal effect on income mobility remains underexplored in China and other developing countries. Most prior studies have focused on educational and occupational mobility, with insufficient attention to long-term income mobility and the heterogeneous mechanisms at play. This study seeks to address this gap.

### 2.2 Theoretical framework and hypotheses

Sociological and economic theories suggest that the Compulsory Education Law (CEL) may influence intergenerational income mobility through three key mechanisms: educational opportunity inequality, occupational opportunity inequality, and differential returns to education. Existing studies have either emphasized the roles of educational opportunities and returns (Bloome et al., 2018) or focused on structural effects and the logic of social stratification and reproduction (Breen, 2010). Building on this literature, we propose three primary mechanisms:

#### 2.2.1 Educational opportunity inequality

Educational expansion policies increase educational supply and lower entry barriers, thereby reducing the constraints of parental socioeconomic status on children’s educational opportunities and promoting intergenerational mobility. According to the MMI and EMI frameworks, while educational opportunities may become more equal as CEL is implemented, disparities in educational quality, school types, and advancement pathways to higher education persist across social classes. Advantaged families leverage their economic and social capital to maintain a competitive edge in the educational process, reinforcing new forms of stratification. Therefore, CEL is likely to reduce educational opportunity inequality at the junior secondary level but may contribute to educational re-stratification at the senior secondary level.

#### 2.2.2 Occupational opportunity inequality

The expansion of educational opportunities enhances upward mobility by improving human capital and expanding access to non-agricultural employment and high-status occupational opportunities, particularly during the transition from agricultural to industrial and service sectors. However, occupational opportunity inequality may persist due to disparities in social capital and networks, as high-socioeconomic-status families remain more likely to obtain managerial and technical positions within the state sector. Thus, while CEL may promote structural mobility, it may also reinforce class-based differentiation in access to high-status occupational opportunities.

#### 2.2.3 Differential returns to education

CEL and related educational expansion policies may alter the structure of returns to education in the labor market. When educational expansion increases the supply of educated labor without a corresponding rise in demand, relative returns to education may decline, thereby fostering greater intergenerational mobility. However, if industrial upgrading raises returns to education while access inequalities persist, intergenerational mobility may be constrained. Additionally, returns to education often vary across social classes, as advantaged families are better able to convert educational attainment into labor market rewards, thereby reinforcing class-based inequality.

The impact of CEL on intergenerational income mobility reflects the combined influence of multiple mechanisms and depends on institutional contexts. The 1986 Compulsory Education Law in China extended 9 years of mandatory schooling and improved access for low-income children through legal and fiscal support. While CEL reduced educational opportunity inequality at the compulsory level, new stratification emerged in senior secondary education. The policy also expanded non-agricultural employment opportunities, yet access to high-status occupations remained concentrated among advantaged families. Given the predominantly rural population, limited educational diversity, and few non-agricultural or high-status occupational opportunities in the 1990s, the positive effects of CEL on intergenerational income mobility were likely more pronounced during this period. Based on this analysis, we propose the following hypotheses:

*H1*: CEL improves intergenerational income mobility in China.

*H2*: CEL enhances mobility by reducing educational opportunity inequality, primarily through its effects at the junior secondary level.

*H3*: CEL promotes mobility by improving access to non-agricultural employment opportunities, while its effects on high-status occupational opportunities remain limited.

*H4*: Class-based differences in returns to education may offset the positive effects of CEL on intergenerational income mobility.

The effects of CEL are likely to vary across population groups. Under China’s dual urban–rural structure, rural areas have long faced systemic constraints, including limited educational resources, weaker teaching capacity, and restricted access to schooling (Golley and Kong, 2018). Consequently, CEL is expected to have a stronger impact on improving educational opportunities for rural children. Regarding gender, traditional son-preference norms have disproportionately constrained girls’ educational attainment, increasing their likelihood of school withdrawal due to educational costs (Dong et al., 2008). The compulsory and universal provisions of CEL are expected to enhance girls’ access to education and improve their intergenerational mobility. However, the extent of these gains may be limited by institutional and social constraints. Based on these considerations, we propose two additional hypotheses:

*H5*: CEL exerts stronger effects on intergenerational mobility for rural children than for their urban counterparts.

*H6*: CEL has stronger effects on intergenerational mobility for women than for men.

## 3 Policy background and research methods

### 3.1 Policy background

On April 12, 1986, China enacted the Compulsory Education Law (CEL), which took effect on July 1 of the same year. This law mandated a nine-year system of universal, free, and compulsory education, comprising 6 years of primary school and 3 years of junior secondary school. It required provincial-level governments, including provinces, autonomous regions, and municipalities, to establish their implementation timelines based on local economic and cultural conditions.

The law required all children who reached the age of six to enroll in school and complete the full nine-year program. In underdeveloped regions, enrollment could be postponed until age seven. Notably, only children younger than 15 during the implementation year were subject to the law, creating a policy exposure cutoff based on birth year. This policy design suggests that individuals born after a province-specific cutoff date (i.e., the implementation year minus 15) were legally required to attend school, thereby increasing their chances of completing junior secondary education. Given that early school leaving was more common among children from disadvantaged backgrounds, the law likely helped raise the educational attainment of this group and reduce the intergenerational transmission of disadvantage. Thus, the reform holds theoretical potential to improve intergenerational income mobility.

A key empirical feature of the policy is its staggered implementation across provinces. While about 10 provinces enacted the law in 1986, another 10 followed in 1987, and the last adopters implemented it by 1994. This temporal variation creates a quasi-experimental setting that allows for difference-in-differences estimation. Specifically, individuals born after the cutoff year in each province form the treatment group, while those born before serve as the control group. For instance, in provinces that enacted the law in 1986, the cutoff cohort is 1971, meaning that those born after September 1971 were affected by the law.

Official statistics from the National Bureau of Statistics indicate that the reform had substantial effects on educational outcomes. The primary-to-junior secondary transition rate rose from 68.4% in 1985 to 90.8% by 1995—an average annual increase of 2.24 percentage points. During the preceding period (1981–1985), the rate remained largely flat, ranging from 66.2 to 68.4%. At the provincial level, except for a few western regions (e.g., Qinghai, Ningxia, Xinjiang), the upward trend closely mirrored national patterns. Additionally, the gross enrollment rate for lower secondary education among the 12–14 age group increased from 66.7% in 1990 to 78.4% in 1995, averaging a 1.17 percentage point increase per year.^1^ A detailed timeline of provincial implementation is presented in Table 1.

**Table 1:** Years of provincial implementation of the CEL in China.

Implementation year	Provinces	Unaffected cohorts	Cutoff year	Affected cohorts	School entry year of affected cohorts
1986	Beijing, Hebei, Shanxi, Liaoning, Heilongjiang, Shanghai, Zhejiang, Jiangxi, Chongqing, Sichuan, Ningxia	≤1970	1971	≥1972	≥1978
1987	Tianjin, Jilin, Jiangsu, Anhui, Shandong, Henan, Hubei, Guangdong, Yunnan	≤1971	1972	≥1973	≥1979
1988	Fujian, Guizhou, Shaanxi, Xinjiang	≤1972	1973	≥1974	≥1980
1989	Inner Mongolia, Qinghai	≤1973	1974	≥1975	≥1981
1991	Gansu	≤1975	1975	≥1977	≥1983
1992	Hunan, Guangxi, Hainan	≤1976	1977	≥1978	≥1984
1994	Tibet	≤1978	1979	≥1980	≥1986

Implementation years are based on official provincial-level documents. For consistency, implementation dates on or after September 1 are coded as starting in the following year. “Unaffected cohorts” are defined as individuals born in or before the cutoff year. “Affected cohorts” are those born in or after the cutoff year. “School entry year” indicates the approximate year that affected cohorts entered formal education.

### 3.2 Research methods

#### 3.2.1 Difference-in-differences model

We use a difference-in-differences identification strategy to estimate the causal impact of the 1986 Compulsory Education Law on intergenerational income mobility. This approach follows Pekkarinen et al. (2009), with subsequent refinements in Betthäuser (2017) and Tang et al. (2020).

##### 3.2.1.1 Direct estimation using a single regression

Since individuals born in the same province and year cohort were uniformly exposed to the policy, we initially estimate the intergenerational income elasticity for each province–cohort cell:


(1) $$Y_{c}=\gamma_{0}+\gamma_{\mathrm{jt}}Y_{p}+\gamma_{2}X+\omega$$

Where $Y_{c}$ denotes the income of the child (measured by income rank percentile), $Y_{p}$ denotes the income of the parent, X is a vector of control variables (including child and parental age (and squared), and survey year dummies), and ω is the error term. The coefficient $\gamma_{\mathrm{jt}}$ captures the intergenerational income elasticity, and $1-\gamma_{\mathrm{jt}}$ reflects income mobility.

Next, we examine the relationship between policy exposure and the intergenerational income elasticity across cohort–province cells:


(2) $$\gamma_{\mathrm{jt}}=\beta_{0}+\beta_{1}\mathrm{CE}L_{\mathrm{jt}}+\varphi D_{j}+\pi D_{t}+\varepsilon_{\mathrm{jt}}$$

Where $\mathrm{CE}L_{\mathrm{jt}}$ denotes the intensity of exposure to the Compulsory Education Law (CEL) for birth cohort t in province j ; $D_{j}$ and $D_{t}$ are province and cohort fixed effects, respectively.

Given the limited sample size in certain province–cohort cells, we adopt a pooled regression specification by substituting Equation 2 into Equation 1, resulting in the following model:^2^


(3) $$\begin{array}{l} Y_{c}=\gamma_{0}+\beta_{0}Y_{p}+\beta_{1}Y_{p}*\mathrm{CE}L_{\mathrm{jt}}+\mathrm{\Omega CE}L_{\mathrm{jt}}+\varphi Y_{p}*D_{j}\\ +\pi Y_{p}*D_{t}+\Psi D_{j}+\Pi D_{t}+\gamma_{2}C+\omega \end{array}$$

Where the interaction term $Y_{p}*\mathrm{CE}L_{\mathrm{jt}}$ captures the heterogeneous effect of the policy on intergenerational income persistence, a negative $\beta_{1}$ indicates that the policy reduced intergenerational income persistence (i.e., enhanced mobility). As in Pekkarinen et al. (2009), we include interactions between parental income and both province and cohort fixed effects to control for heterogeneous baseline trends. C includes additional controls: child and parental age (and squared), child gender, and child hukou status (hukou). Standard errors are clustered at the province level.

##### 3.2.1.2 Extended specification: controlling for province-specific cohort trends

To account for long-term, province-specific cohort trends that may confound the estimated policy effect, we follow Betthäuser (2017) and Tang et al. (2020) by adding province-cohort fixed effects.


(4) $$\begin{array}{l} Y_{c}=\gamma_{0}+\beta_{0}Y_{p}+\beta_{1}Y_{p}*\mathrm{CE}L_{\mathrm{jt}}+\mathrm{\Omega CE}L_{\mathrm{jt}}+\varphi Y_{p}*D_{j}\\ +\pi Y_{p}*D_{t}+\Psi D_{j}+\Pi D_{t}+ΤD_{j}*D_{t}+\gamma_{2}C+\omega \end{array}$$

This specification ensures that any unobserved linear trends unique to each province–cohort pair are properly controlled.

#### 3.2.2 Correction for income measurement bias

Intergenerational income mobility estimates may suffer from two important issues: the lifecycle bias and attenuation bias. While both affect the accurate measurement of permanent income, they arise from different mechanisms: lifecycle bias reflects systematic errors when income is observed at ages that are too early or too late in the life cycle, whereas attenuation bias results mainly from random shocks or reporting errors. For brevity, we refer to both as “income measurement bias.”

Following Fan et al. (2021), we apply three adjustments. First, age and age-squared terms for both parents and children are included to reduce lifecycle bias. Second, children are observed at around age 30 (ensuring they have left school and are established in the labor market), while parents are observed below age 60 (avoiding retirement effects), ensuring comparability across generations. Third, we adopt the rank–rank method, which is less sensitive to income shocks and age differences than log–log regressions. Prior studies (Nybom and Stuhler, 2017; Deutscher and Mazumder, 2023) show that income ranks remain relatively stable across the life cycle, making them a useful proxy for permanent income.

By defining comparable age intervals and employing the rank–rank approach, we mitigate measurement bias and obtain estimates that more closely reflect the intergenerational transmission of permanent income and relative income position.

#### 3.2.3 Correction for co-residence bias

Co-residence selection bias occurs in household survey data because non-co-resident adult children are often missing, leading to biased estimates (Emran et al., 2018). To address this issue, we apply a Heckman sample selection model, which corrects for potential non-random selection into the co-resident sample (Yuan and Liu, 2022). The selection equation is specified as follows:


(5) $$P_{i}=\pi_{0}+\pi_{1}Z_{i}+\pi_{2}C_{i}+v_{i}$$

Where $P_{i}$ is a dummy equal to 1 if the child is co-residing with the household head and 0 otherwise. $Z_{i}$ is an exclusion restriction, measured by marital status (married = 1), which affects co-residence likelihood but is not directly related to income. $C_{i}$ includes child and parental age (and squared), child gender, years of schooling, household type (urban/rural), and region fixed effects.

Since the CHIP 1995 and 2002 surveys did not collect information on non-co-resident children of household heads, while the 2007 survey did, we follow Yang and Zhang (2015) and use the 2007 data to compute the inverse Mills ratio (IMR) for the co-resident sample. The rationale is twofold. First, the core determinants of co-residence—such as marriage, age, rural–urban hukou status, and education—are expected to have remained broadly stable between 1995 and 2007. Second, given that the study sample is primarily rural, the high prevalence of co-resident children in 1995 and 2002 suggests weaker selection concerns for earlier cohorts compared with 2007. In addition, we perform robustness checks, including entropy balancing and propensity score reweighting, to ensure the reliability of the results. The resulting IMR, estimated from Equation 5, is then included as a control in Equations 3, 4, thereby correcting for potential selection bias.

## 4 Data and variables

### 4.1 Data source

This study uses data from the China Household Income Project (CHIP), a nationally representative household survey coordinated by the China Institute for Income Distribution at Beijing Normal University in collaboration with partner institutions. CHIP employs a stratified random sampling design and has been conducted in six waves (1988, 1995, 2002, 2007, 2013, and 2018), collecting detailed information on demographics, employment, earnings, and household income, expenditure, and assets. Because income measures in 1988 and 2007 are not fully consistent with those in other years, we restrict our analysis to the 1995, 2002, 2013, and 2018 waves.

Compared with the China Family Panel Studies (CFPS, 2010–2022) and the China Health and Nutrition Survey (CHNS, 1989–2021), both widely used in intergenerational mobility research, CHIP provides a longer time span and covers earlier birth cohorts, which is essential for analyzing individuals born around 1970. In addition, unlike CHNS, CHIP collects information on non-co-resident adult children, which helps us examine potential co-residence selection bias. These features make CHIP particularly well-suited to this study.

### 4.2 Sample construction

We construct the analytical sample through a two-stage process. In the first stage, we establish intergenerational links using household relationship rosters. We create two types of parent–child pairs: upward links, where the household head or spouse is treated as the child and connected to their parents (representing the parental generation), and downward links, where the children of the household head are defined as the child generation, with the head and spouse representing the parental generation.

In the second stage, we implement a comprehensive set of sample restrictions. The child generation is defined as individuals aged 23 to 40 at the time of the survey, ensuring adequate labor market experience while avoiding retirement-related distortions. The lower bound of 23 reflects the typical age by which individuals have completed schooling and entered the labor market. The upper bound of 40 enhances comparability across cohorts while maintaining sufficient sample size, noting that observations above age 40 are relatively scarce. We exclude cases where the parent–child age gap is less than 16 years and cases in which the parental generation is older than 60. Since 60 is commonly regarded as the retirement age, this restriction ensures that the parental generation remains within the working-age range, allowing for valid measures of labor income. We also drop observations with missing or implausible values in key variables such as income and education.

Finally, we retain only individuals born in 1960 or later, as earlier cohorts are sparsely represented in the data. To avoid potential confounding effects of the higher education expansion in 1999, we further restrict the sample to those born before 1980.

Table 2 presents the distribution of birth cohorts by provincial implementation years of the Compulsory Education Law (CEL), with the bolded cells denoting the cohorts exposed to the law.^3^

**Table 2:** Sample distribution of birth cohort by year of CEL implementation.

Birth cohort	Year of CEL						Total
	1986	1987	1988	1989	1991	1992	
1960–1964	22	35	2	0	9	2	70
1965–1969	222	261	31	0	37	21	572
1970	133	184	22	0	20	7	366
1971	**181**	232	32	0	25	17	487
1972	**243**	**275**	37	0	36	34	625
1973	**51**	**39**	**13**	0	8	6	117
1974	**67**	**62**	**22**	**0**	8	16	175
1975	**76**	**87**	**27**	**0**	3	24	217
1976	**108**	**107**	**34**	**0**	**12**	23	284
1977	**120**	**160**	**40**	**0**	**12**	**22**	354
1978	**140**	**205**	**44**	**0**	**19**	**40**	448
1979	**160**	**202**	**47**	**0**	**16**	**49**	474
1980–1984	**82**	**293**	**0**	**1**	**25**	**48**	449
1985–1989	**369**	**1041**	**0**	**5**	**78**	**105**	1,598
1990–1994	**289**	**815**	**0**	**31**	**71**	**66**	1,272
1995–1999	**43**	**129**	**0**	**16**	**3**	**6**	197
Total	2,306	4,127	351	53	382	486	7,705

Bolded cells indicate cohorts exposed to the CEL in each province. The sample is drawn from the 1995, 2002, 2013, and 2018 CHIP waves, restricted to individuals aged 23–40.

For policy evaluation, the sample was further refined as follows. First, to avoid sample size imbalances across policy periods, we excluded Inner Mongolia, which implemented the Compulsory Education Law in 1989. Second, to ensure comparability across cohorts, we excluded individuals born after 1976 and provinces implementing the law in 1991 or 1992.^4^ The final analytical sample consists of 2,258 valid intergenerational observations, which centers on birth cohorts directly exposed to provincial variations in CEL implementation. Given the birth cohorts under study and the ages at which their incomes are observed, the main empirical analyses rely on the 1995 and 2002 waves. Although the 2013 and 2018 waves are not included in the baseline regressions, they are included only for descriptive purposes in the data section, as shown in Table 2.

### 4.3 Variables

#### 4.3.1 Income

The dependent variable is individual labor income, defined as the total of wage income and net business income. Net business income includes both agricultural operating income and non-agricultural self-employment income. In rural samples, agricultural business income is reported at the household level, which we allocate to individuals according to their share of household farm labor time. Following Wang and Shao (2021), household agricultural net income is calculated as agricultural revenue minus production costs and then allocated to individuals in proportion to their farm labor time within the household.

Missing values for household agricultural income or farm labor time are very rare (less than 0.5%). When they occur, we drop these cases rather than impute values to avoid bias. Negative agricultural income accounts for only 0.4% of cases, so we restrict the analysis to non-negative observations. Compared with treatments of agricultural income in CFPS and CHNS, the labor-time allocation method yields a reasonable measure of individual agricultural income (Yuan and Liu, 2022).

We deflate all income values from the 1995, 2002, and 2013 CHIP waves to 2018 constant prices using province-level Consumer Price Index (CPI) data. To minimize measurement error and reduce the influence of outliers, we represent income in the regressions by income rank, that is, percentile position in the distribution. We measure parental income as the average of the father’s and mother’s income, since both parents contribute to household resources and the combined measure better reflects family socioeconomic status (Beller, 2009). In subgroup and heterogeneity analyses, we recalculate income ranks within each group to ensure comparability across groups.

#### 4.3.2 CEL

The core explanatory variable is exposure to the Compulsory Education Law (CEL), which varies by province and birth cohort. Using the implementation years shown in Table 1, the literature typically adopts three approaches to measure the policy variable:^5^

1. Binary treatment indicator. Individuals are assigned a value of 0 if they were born before the provincial implementation cutoff year (i.e., those older than 15 at the time of implementation), and 1 if born in or after the cutoff year. This specification is intuitive but overlooks heterogeneous exposure across different schooling ages.
2. Continuous exposure measures capture the intensity of policy exposure. Two typical forms are:

- Standardized exposure. Those aged 15 or older at implementation are coded as 0 (no exposure), those younger than 6 as 1 (full exposure), and those aged 6–15 take intermediate values as specified in Equation 6.

(6) $$\mathrm{CE}L_{\mathrm{jt}}=(16-(P_{j}-B_{t}))/9$$

Where $P_{j}$ denotes the year of policy implementation in province j , and $B_{t}$ is the birth year of the child. $\mathrm{CE}L_{\mathrm{jt}}$ measures the degree of policy exposure. Here, 16 denotes the completion age of compulsory education; $\left(P_{j}-B_{t}\right)$ is the child’s age when the policy was implemented; $\left(16-(P_{j}-B_{t})\right)$ represents the remaining years of compulsory schooling available. Dividing by 9, the full length of compulsory schooling, normalizes the measure into the [0,1] range, representing the proportion of compulsory schooling that an individual could still receive at the time of policy implementation (e.g., Chen et al., 2021).

- Years of exposure. Alternatively, researchers may use $16-(P_{j}-B_{t})$ to measure the number of school years affected (e.g., Zuo et al., 2023). Both continuous exposure variables capture the intensity of treatment, that is, the degree of policy exposure, and represent the mainstream approach in the current literature.

1. Provincial policy intensity. These indicators measure the strength of CEL reform at the provincial level using pre-reform educational conditions and contemporaneous changes in investment or accessibility. Typical measures include the share of the population with fewer than nine years of schooling before implementation, increases in per capita education expenditure, or changes in junior secondary enrollment rates. These indicators are typically interacted with exposure measures to proxy policy implementation intensity and to capture heterogeneity in treatment effects (Huang, 2015; Zhang and Zhao, 2023). While this approach emphasizes cross-province differences, it may introduce measurement error due to confounding external conditions.

In the baseline analysis, we adopt the standardized exposure measure. Robustness checks also include the binary indicator, the years of exposure, and interactions with provincial intensity proxies to examine heterogeneous reform strength.^6^

Finally, in defining exposure, we follow the intention-to-treat (ITT) approach commonly adopted in the literature, rather than relying on individuals’ actual schooling ages or years of education, to mitigate endogeneity concerns from educational choices (Xiao et al., 2017; Tang et al., 2020). Thus, the treatment variable indicates whether a cohort was eligible for the CEL based on its provincial rollout year, irrespective of compliance or enforcement timing. Accordingly, our estimates should be interpreted as ITT effects, capturing the average impact of policy exposure rather than the exact effect of individual schooling responses.

#### 4.3.3 Control variables

Key control variables include gender (male = 1, female = 0), hukou registration status (non-agricultural = 1, agricultural = 0), marital status (married = 1, unmarried = 0), household type (urban = 1, rural = 0), and the current province of residence.

Since CHIP does not record the province where individuals received compulsory education, we follow existing literature by assuming that the province of education corresponds with the current province of residence. This assumption may introduce a misclassification error. To assess its extent, we draw on the 2018 CHIP wave, which records the hukou registration province at age 14. Results indicate that only about 2% of individuals’ hukou registration province at age 14 differed from their current residence. Given historically lower inter-provincial migration rates, this percentage is likely even smaller in earlier survey years. Therefore, any potential bias introduced by this assumption is expected to be minimal.

### 4.4 Descriptive statistics

Table 3 presents the descriptive statistics for all variables employed in the analysis. The average age of the children’s cohort is 26 years. Their mean annual income is 6,193 RMB, and the average years of schooling is 8.5. Within the sample, 71% are male, 53% are married, 15% hold a non-agricultural hukou, and 16% live in urban households. About 47% of the sample resides in eastern provinces.^7^ Approximately 50% of the sample was affected by the Compulsory Education Law (CEL), with an average exposure intensity of 0.12 and an average of 1.18 affected semesters. The parental generation has a mean age of 52 years and an average annual income of 5,496 RMB.

**Table 3:** Descriptive statistics for key variables (*N* = 2,258).

Variables	Unit	Mean	SD	Min	Max
Age	Years	26.18	2.85	23	36
Annual income	RMB	6,193	8,545	22	170,000
Years of schooling	Years	8.49	2.91	0	19
Male	Dummy (1 = Yes)	0.71	0.46	0	1
Married	Dummy (1 = Yes)	0.53	0.50	0	1
Non-agricultural hukou	Dummy (1 = Yes)	0.15	0.36	0	1
Urban household	Dummy (1 = Yes)	0.16	0.36	0	1
Eastern region	Dummy (1 = Affected)	0.47	0.50	0	1
CEL binary indicator	Dummy (1 = Yes)	0.50	0.50	0	1
CEL-standardized exposure	Index (0–1)	0.12	0.16	0	0.56
CEL-year of exposure	Semesters	1.18	2.16	0	8
Average parental age	Years	51.91	3.74	40.50	60
Average parental income	RMB	5,496	5,940	28	92,070

Data are sourced from the CHIP. The sample includes individuals aged 23 to 40 who were born between 1966 and 1975.

Table 4 presents intergenerational income transition matrices for cohorts born before and after the implementation of the CEL. Treatment status is defined as a binary indicator equal to 1 if the individual was exposed to the law and 0 otherwise. Labor incomes for both generations are categorized into quintiles of equal size, which is appropriate given the moderate sample size. Three key findings emerge from the analysis:

- Substantial income persistence is observed in both pre- and post-policy cohorts (Panels A and B), indicating that income ranks are strongly correlated with their parental background regardless of policy exposure.
- Panel C reports changes in transition probabilities (post-policy minus pre-policy). An overall decline in diagonal probabilities, especially in the bottom two quintiles, indicates a reduction in intergenerational income persistence and a corresponding improvement in mobility. Although two diagonal cells increase slightly, their magnitudes are modest and do not affect the broader mobility-enhancing trend.
- Upward mobility improves for disadvantaged groups. The most significant positive changes are observed in the upper-right cells of Panel C, especially among individuals from the lowest parental income quintile. Their increased likelihood of advancing to higher income quintiles suggests that CEL exerted substantive equalizing effects.

**Table 4:** Intergenerational income transition matrices.

Parental income quintile	Child income quintile					Observations
	1st	2nd	3rd	4th	5th	
Panel A: pre-CEL group (unaffected by the law)						
1st	0.522	0.217	0.093	0.080	0.088	226
2nd	0.170	0.359	0.287	0.090	0.094	223
3rd	0.138	0.223	0.299	0.214	0.125	224
4th	0.107	0.129	0.205	0.330	0.228	224
5th	0.063	0.071	0.116	0.286	0.464	224
Panel B: post-CEL group (affected by the law)						
1st	0.417	0.237	0.127	0.123	0.096	228
2nd	0.197	0.386	0.184	0.127	0.105	228
3rd	0.154	0.181	0.344	0.172	0.150	227
4th	0.145	0.150	0.225	0.291	0.189	227
5th	0.088	0.044	0.123	0.286	0.458	227
Panel C: differences in transition probabilities (post – pre)						
1st	−0.105	0.020	0.034	0.043	0.008	—
2nd	0.027	0.027	−0.103	0.038	0.011	—
3rd	0.016	−0.043	0.045	−0.042	0.025	—
4th	0.038	0.020	0.019	−0.040	−0.038	—
5th	0.026	−0.027	0.007	0.001	−0.006	—

Each panel reports the conditional probability of a child being in a given income quintile, conditional on the parental quintile. Panel C presents the difference in transition probabilities between cohorts affected by the Compulsory Education Law (post-CEL) and those unaffected (pre-CEL).

## 5 Empirical results

### 5.1 Baseline results

Table 5 presents the baseline estimates of the Compulsory Education Law (CEL) on intergenerational income mobility. Three model specifications are reported, corresponding to the empirical strategies discussed in Section 3.2. Column 1 implements a reduced-form version of Equation 3, omitting interaction terms between parental income and province or cohort fixed effects. Column 2 reports the complete one-step model, which includes all interaction terms to account for regional and temporal heterogeneity. Column 3 extends this by incorporating province-by-cohort fixed effects, as in Equation 4, to flexibly account for province-specific linear cohort trends. Following Chen et al. (2021), birth years are grouped into three cohort bins: 1965–1969, 1970–1974, and 1975, ensuring sufficient within-group variation.^8^

**Table 5:** Baseline results.

	Reduced form	One-step model	With linear trends
	(1)	(2)	(3)
Parental income	0.372***	0.065	0.132*
	(0.036)	(0.052)	(0.063)
CEL	22.483*	28.500**	24.475**
	(11.007)	(10.673)	(11.494)
Parental income × CEL	−0.315**	−0.459**	−0.463**
	(0.146)	(0.199)	(0.201)
Inverse Mills ratio	3.710	3.851	4.052
	(4.053)	(4.098)	(4.094)
Parental income × (province/cohort)	×	√	√
Province × cohort fixed effects	×	×	√
Observations	2,258	2,258	2,258
R^2^	0.303	0.313	0.326

Robust standard errors clustered by province in parentheses. ***p < 0.01, **p < 0.05, *p < 0.10. All regressions include controls for child and parental age (and squared), child gender, hukou status, household type, survey year, and province and cohort fixed effects. CEL denotes exposure to the CEL.

Across all specifications, the interaction term between parental income and CEL exposure is negative and statistically significant at the 5% level, indicating a reduction in intergenerational income persistence. This effect is largely consistent across different model specifications, including those accounting for regional and cohort heterogeneity (Column 2) and cohort-specific trends at the provincial level (Column 3), suggesting that the implementation of CEL contributed to higher intergenerational income mobility.

In Column 3, the coefficient of the interaction term is −0.463, indicating that a 0.1-unit increase in CEL exposure reduces the intergenerational rank correlation by 0.0463 on average. At the sample mean of CEL intensity (0.12; see Table 3), the rank correlation declines from 0.132 to 0.076 (0.132–0.463 × 0.12 = 0.076), implying a roughly 42% reduction in intergenerational persistence and a substantial increase in mobility. It should be noted that this estimate captures the short-term average policy effect, as CEL exposure in our analysis ranges from 0 to 0.56 rather than 0 to 1. The relatively low baseline rank–rank correlation reflects its calculation within province–birth cohort cells and the inclusion of province fixed effects and cohort-specific linear trends; however, since our focus is on the magnitude and direction of the policy effect rather than the absolute mobility level, this does not affect the validity of our conclusions.

The coefficient on the inverse Mills ratio is statistically insignificant across all models, indicating that co-residence selection bias, which is an inherent concern when constructing parent–child links from household rosters, is unlikely to affect the estimates. This finding is consistent with the demographic context of China in the 1990s and early 2000s, when co-residence between adult children and parents remained common among the studied cohorts (born 1966–1975). Such demographic patterns further mitigate potential selection concerns.

### 5.2 Parallel trends test

The validity of the difference-in-differences approach relies on the parallel trends assumption: in the absence of the Compulsory Education Law (CEL), early- and late-adopting provinces would have experienced similar intergenerational income mobility trends across birth cohorts. While the counterfactual cannot be observed directly, we assess its plausibility by examining pre-policy trends.

Since our baseline coefficient of interest is the interaction between parental income and CEL exposure, we estimate a triple-difference model to test whether pre-policy mobility trends differed between early- and late-adopting provinces, as specified in Equation 7:


(7) $$\begin{array}{l} Y_{c}=\gamma_{01}+\beta_{01}Y_{p}+\beta_{11}Y_{p}*Pe_{t}*Tr_{j}+\varphi_{1}Y_{p}*Tr_{j}\\ +\pi_{1}Y_{p}*Pe_{t}+\Psi_{1}Tr_{j}+\Pi_{1}Pe_{t}+{Τ}_{1}Tr_{j}*Pe_{t}+\gamma_{21}C+\omega_{1} \end{array}$$

Here, $Pe_{t}$ is a dummy for the birth cohort group preceding the implementation year. $Tr_{j}$ indicates treatment provinces (those that adopted CEL in 1987 or 1988). $Y_{p}*Pe_{t}*Tr_{j}$ is the triple interaction capturing differences in intergenerational mobility trends across provinces over pre-policy birth cohorts. $\beta_{1}$ is the coefficient of interest, while a statistically insignificant estimate would suggest no pre-existing differential trends in intergenerational mobility between treated and control provinces, thereby validating the parallel trends assumption.

The analysis is restricted to pre-policy cohorts—individuals aged 15 or older when CEL was implemented in their province of residence. Following Xu and Zhong (2022), we group birth cohorts into $Pe_{-1}$ (those born 1–3 years before the province-level CEL implementation year) and $Pe_{-2}$ (those born 4–7 years prior). We also conduct robustness checks using alternative time windows surrounding the CEL implementation threshold. Estimation results are reported in Table 6. Across specifications (Columns 1–3), coefficients for the triple interaction are statistically insignificant, indicating no systematic differences in pre-policy mobility trends. Therefore, the parallel trends assumption is empirically supported.

**Table 6:** Parallel trends test.

	Pre-policy: −7 to −1	Pre-policy: −6 to −1	Pre-policy: −5 to −1
	(1)	(2)	(3)
Parental income	0.482***	0.482***	0.477***
	(0.055)	(0.055)	(0.056)
Parental income× $Pe_{-1}\times Tr_{1987}$	−0.119	−0.120	−0.120
	(0.078)	(0.078)	(0.086)
Parental income× $Pe_{-1}\times Tr_{1988}$	−0.078	−0.136	−0.005
	(0.102)	(0.124)	(0.157)
Parental income× $Pe_{t}$	√	√	√
Parental income× $Tr_{j}$	√	√	√
$Pe_{t}*Tr_{j}$	√	√	√
Observations	1,121	1,119	1,083
R^2^	0.298	0.299	0.293

Standard errors are clustered by province in parentheses. ***p < 0.01, **p < 0.05, *p < 0.10. All models control for child and parental age (and squared), child gender, hukou status, survey year dummies, the inverse Mills ratio, province, and birth cohort fixed effects, province trends. “ ${\mathrm{Pe}}_{-1}$ ” includes cohorts born 1–3 years before CEL implementation; “Tr1987” and “ ${\mathrm{Tr}}_{1988}$ ” denote provinces that adopted CEL in those years.

**Figure 1:**
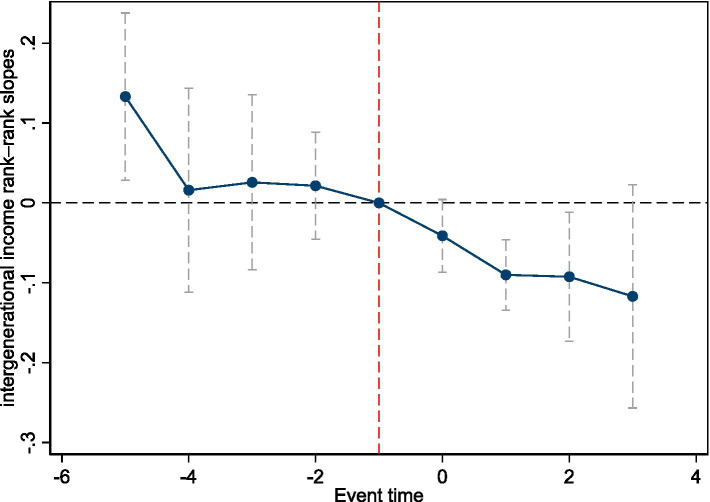
Event-study estimates: parallel trends test. Notes: 90% confidence intervals are shown.

We further assess the parallel trends assumption using an event-study framework. The event window spans 5 years before to 3 years after implementation, with the year immediately preceding the reform as the reference (see Figure 1). Pre-reform coefficients (−4 to −1) are statistically insignificant, confirming the parallel trends assumption (the large and significant coefficient at −5 reflects a small sample size rather than a true deviation). Post-reform coefficients are mostly insignificant, which differs from the DID estimates. A plausible explanation is the staggered provincial rollout of CEL over a short interval (1986–1988), which may attenuate dynamic effects. Nevertheless, this does not undermine our focus on the average treatment effect, as the DID and event-study capture distinct aspects of policy impact. The negative and downward pattern of the coefficients is consistent with the average treatment effect identified in the DID estimates.

### 5.3 Robustness checks

To assess the robustness of our baseline findings, we conduct a comprehensive battery of sensitivity analyses along five dimensions, with results summarized in Table 7.

**Table 7:** Robustness checks.

	Reduced form	One-step model	With linear trends
	(1)	(2)	(3)
A. Adjusted sample range			
1. Birth cohorts 1967–1974	−0.425**	−0.493**	−0.494**
	(0.183)	(0.196)	(0.201)
2. Aged 11–18 at policy implementation	−0.237**	−0.430**	−0.441**
	(0.112)	(0.177)	(0.184)
3. Aged 12–17 at policy implementation	−0.291**	−0.508**	−0.513**
	(0.123)	(0.193)	(0.196)
4. Drop cohorts aged 15 at policy implementation	−0.315**	−0.459**	−0.463**
	(0.146)	(0.199)	(0.201)
B. Contemporaneous policy and confounding controls			
5. Controlling for the Cultural Revolution	−0.315**	−0.459**	−0.463*
	(0.146)	(0.199)	(0.201)
6. Pre-reform provincial characteristics × cohort	−0.373**	−0.444**	−0.441**
	(0.147)	(0.202)	(0.204)
C. Alternative policy measures			
7. Binary treatment indicator (age <15)	−0.078*	−0.078*	−0.074
	(0.041)	(0.044)	(0.045)
8. Year of exposure	−0.022*	−0.032*	−0.034*
	(0.011)	(0.017)	(0.017)
9. provincial policy intensity × exposure	−1.613*	−1.714**	−1.840**
	(0.776)	(0.683)	(0.645)
D. Alternative income specifications			
10. Max parental income	−0.235	−0.381**	−0.373**
	(0.149)	(0.152)	(0.155)
11. Father’s income only	−0.231	−0.372**	−0.367**
	(0.166)	(0.164)	(0.164)
12. Income measured in logs	−0.355*	−0.476*	−0.492*
	(0.176)	(0.247)	(0.247)
E. Sample selection bias			
13. Clustering at the province × cohort	−0.315**	−0.459**	−0.463**
	(0.123)	(0.184)	(0.189)
14. Entropy balancing	−0.314**	−0.456**	−0.459**
	(0.146)	(0.200)	(0.201)
15. Propensity score weighting	−0.321**	−0.460**	−0.463**
	(0.143)	(0.195)	(0.197)

This table presents the coefficients on the interaction term between parental income and CEL across various robustness specifications. Columns (1)–(3) correspond to the baseline models in Table 5. Robust standard errors are clustered by province in parentheses (except for Row 13). ***p < 0.01, **p < 0.05, *p < 0.10.

#### 5.3.1 Adjusted sample range

First, we refine the sample to enhance identification by narrowing the birth cohort to 1967–1974 and restricting exposure to ages 11–18 or 12–17 at the time of the Compulsory Education Law (CEL) implementation, to ensure plausible exposure to the policy. Across all specifications, the interaction term remains negative and statistically significant, indicating that CEL significantly enhanced intergenerational income mobility (Rows 1–3). Second, because cohort classification is by birth year rather than month, we further exclude individuals who were exactly 15 years old at the time of provincial CEL implementation (born 1971–1973) to mitigate potential misclassification. The results remain robust (Row 4).

#### 5.3.2 Excluding contemporaneous policy and confounders

Contemporaneous reforms such as higher-education expansion, free compulsory education, and the National “87” Poverty Alleviation Program (1994) do not affect cohorts born 1966–1975. The principal potential confounder is the Cultural Revolution, which affected cohorts born 1949–1969 (Zhang et al., 2007; Meng and Zhao, 2017); we therefore include a dummy for Cultural Revolution exposure, and the results remain robust (Row 5). In addition, although Column 3 already incorporates province × cohort fixed effects to absorb province-level cohort shocks, we additionally control for interactions between birth cohorts and pre-reform provincial characteristics to mitigate remaining confounding. Following Zuo et al. (2023), these controls are the 1980–1984 averages of per-capita GDP, total population, fiscal balance ratio, and urbanization rate. Across these specifications, the parental income × CEL interaction remains negative and statistically significant, confirming the robustness of the baseline finding (Row 6).

#### 5.3.3 Alternative policy variable

We re-estimate the models using three alternative operationalizations of CEL, as introduced in Section 4.3.2: a binary indicator based on the age-15 cutoff, years of exposure, and the interaction between provincial policy intensity and individual exposure. Across all specifications, the parental income × CEL interaction remains negative, indicating that CEL consistently enhances intergenerational income mobility (Rows 7–9). Moreover, longer exposure and higher policy intensity are associated with larger reductions in intergenerational income persistence, underscoring both the robustness of the baseline finding and the importance of accounting for provincial heterogeneity in policy implementation.

#### 5.3.4 Alternative income specifications

We examine alternative measures of parental income. Whether defined as the father’s income or the higher income of the two parents, the interaction with CEL remains negative and statistically significant (Rows 10–11), demonstrating robustness to income definitions. In addition, since prior studies commonly use log income, we replace income ranks with log parental income, applying a 1% two-sided trimming to address outliers. Table 7, Row 12 shows that the interaction between parental income and CEL remains significantly negative, confirming the robustness of our results.

#### 5.3.5 Sample selection bias

First, we cluster standard errors at the province × cohort level to account for limited sample sizes, and the baseline findings remain robust (Row 13). Second, to assess the validity of extending the 2007 selection equation to other periods, we reweight the 2007 sample using entropy balancing and propensity score matching to align with earlier covariate distributions. We then re-estimate the selection equation to compute updated inverse Mills ratios. Both methods yield interaction coefficients consistent with the baseline, indicating that cross-period assumptions do not compromise the results (Rows 14–15).

### 5.4 Mechanism analysis

The baseline regression results indicate that the Compulsory Education Law (CEL) has significantly promoted intergenerational income mobility. To further explore the potential mechanisms, this paper examines three dimensions: educational opportunity inequality, occupational opportunity inequality, and differential returns to education.

#### 5.4.1 Educational opportunity inequality

This study measures educational opportunity inequality using indicators of intergenerational educational mobility. The regression model follows Equation 4, where the dependent variable is the child’s educational attainment, measured by years of schooling, completion of junior and senior secondary schooling. Key explanatory variables include parental years of schooling and senior secondary completion, and their interactions with the Compulsory Education Law (CEL). All other controls are consistent with Equation 4, except parental age and its squared term. Ordinary least squares (OLS) is applied to continuous outcomes, and logit models to binary outcomes.

Table 8 reports the results. The interaction between parental education and CEL in Column (1) is insignificant, indicating no overall effect on educational opportunity inequality. At different schooling levels (Columns 2–3), CEL significantly weakened the association between parental education and junior secondary completion but strengthened that with high school completion, implying less inequality at the compulsory stage but greater inequality thereafter. Replacing parental education with parental income rank (Columns 4–5) yields consistent results, confirming robustness.

**Table 8:** Mechanism analysis: educational opportunity inequality.

	Years of schooling	Junior sec.	Senior sec.	Junior sec.	Senior sec.
	(1)	(2)	(3)	(4)	(5)
Parental schooling × CEL	0.012				
	(0.197)				
Parental high school × CEL		−2.877**	3.706**		
		(1.316)	(1.835)		
Parental income rank × CEL				−0.012*	0.048***
				(0.007)	(0.009)
Inverse Mills ratio	6.112***	0.940**	4.726***	0.281	2.623***
	(0.440)	(0.435)	(0.679)	(0.303)	(0.266)
Observations	2,258	2,235	2,225	2,258	2,258
R^2^ / Pseudo R^2^	0.543	0.157	0.379	0.129	0.335

Robust standard errors clustered by province in parentheses. ***p < 0.01, **p < 0.05, *p < 0.1. All models follow the specification of Equation 4. Column (1) is estimated by OLS; Columns (2)–(5) are estimated by logit models. Controls: CEL, gender, hukou, age, age^2^, birth cohort, province, province trends, parental schooling (in column 1), parental high school (in columns 2–3), parental income rank (in columns 4–5).

These findings confirm the stage-specific impact of CEL, consistent with Hypothesis 2. At the junior secondary level, the policy broadened access for students from disadvantaged backgrounds. At the senior level, differences in family resources reinforced educational stratification. The results align with the Maximally Maintained Inequality (MMI) and Effectively Maintained Inequality (EMI) frameworks. While greater stratification at higher levels may have limited intergenerational mobility, equalization in compulsory education remained the primary driver of overall mobility gains.

#### 5.4.2 Occupational opportunity inequality

The dataset lacks sufficient parental occupation information, which prevents direct estimation of intergenerational occupational mobility. To address this limitation, we proxy parental socioeconomic status (SES) using parental education and income rank, and measure children’s occupational inequality along two dimensions. The first is non-agricultural employment, indicating whether individuals moved from low-income agricultural work into industrial or service sectors, capturing structural mobility during China’s economic transition. The second is high-status occupations, defined as holding a managerial or professional positions (unit or department head, professional staff) within the state sector, including government agencies, public institutions, and state-owned or state-controlled enterprises. The model specification follows that in Section 5.4.1.

Table 9 reports the results. Columns (1)–(2) show that the Compulsory Education Law (CEL) significantly weakened the association between non-agricultural employment and parental SES, indicating improved equality of occupational opportunity. By expanding access to basic education, CEL enhanced the human capital of children from disadvantaged families, thereby increasing their likelihood of entering non-agricultural sectors and improving their income prospects.

**Table 9:** Mechanism analysis: occupational opportunity inequality.

	Non-agricultural employment		High-status occupations	
	(1)	(2)	(3)	(4)
Parental education × CEL	−0.467**		3.801*	
	(0.202)		(2.090)	
Parental income rank × CEL		−0.035**		0.127*
		(0.014)		(0.069)
Inverse Mills ratio	0.299	−0.032	1.533	1.170
	(0.523)	(0.468)	(0.789)	(0.736)
Observations	1,695	1,695	1,665	1,763
Pseudo R^2^	0.175	0.182	0.287	0.341

Robust standard errors clustered by province in parentheses. ***p < 0.01, **p < 0.05, *p < 0.1. All models follow the specification of Equation 4 and are estimated using logit regressions. Controls: CEL, gender, hukou, age, age^2^, birth cohort, province, province trends, parental education (only in columns 1, 3), parental income rank (only in columns 2, 4).

In contrast, Columns (3)–(4) indicate that CEL strengthened the association between high-status occupational attainment and parental SES, suggesting increased inequality in access to elite occupations. This pattern reflects a cumulative advantage process, whereby stratification at the senior secondary level and differences in family resources translated into advantages in obtaining high-status positions. The resulting occupational disparities partially offset the positive effects of CEL on intergenerational income mobility.

#### 5.4.3 Differential returns to education

Theoretical research suggests that higher returns to education, even under unchanged educational opportunity, may reduce intergenerational income mobility. To test this mechanism, we estimate the effect of the Compulsory Education Law (CEL) on the marginal returns to education. The dependent variable is log labor income, and the key explanatory variable is the interaction between years of schooling and CEL. Control variables include years of schooling, CEL, gender, hukou, age and its square (proxying work experience), birth cohort, province fixed effects, province-specific trends, and the inverse Mills ratio. As shown in Column (1) of Table 10, CEL does not have a significant effect on the average returns to education.

**Table 10:** Mechanism analysis: differential returns to education.

	Full sample	Low-income parents	High-income parents	Full sample
	(1)	(2)	(3)	(4)
Years of schooling × CEL	0.062	−0.022	0.246*	
	(0.136)	(0.185)	(0.125)	
Years of schooling × CEL × Parental income status				0.191*
				(0.101)
Inverse Mills ratio	0.027	−0.063	0.130	−0.025
	(0.171)	(0.279)	(0.172)	(0.167)
Observations	2,258	1,129	1,129	2,258
R^2^	0.229	0.157	0.225	0.254

Robust standard errors clustered by province in parentheses. ***p < 0.01, **p < 0.05, *p < 0.1. Columns (1)–(4) estimate Equation 4 with log income; Controls: CEL, years of schooling, gender, hukou, age, age^2^, birth cohort, province, province trends, two-way interaction terms (only in column 4).

Building on earlier evidence of increased educational and occupational inequality, we further examine whether the effects of CEL on returns to education vary by socioeconomic background. The sample is split by parental income status (above or below the mean). Columns (2)–(3) indicate that CEL significantly increased the returns to education among individuals from high-income families, but no significant effect is observed among those from low-income families.

To formally assess this heterogeneity, we introduce a triple interaction term (years of schooling × CEL × parental income status). The positive and significant coefficient (0.191) in Column (4) indicates that CEL increased returns to education disproportionately among individuals from high-income families. This divergence implies that CEL indirectly intensified social stratification through unequal returns to education, thereby constraining intergenerational income mobility.

### 5.5 Heterogeneous effects

This section examines the heterogeneous effects of the Compulsory Education Law (CEL) on intergenerational income mobility across hukou status and gender. Specifically, we extend Equation 4 by including a triple interaction term between parental income, CEL, and the heterogeneity variable, while simultaneously controlling for all corresponding two-way interactions. The estimation results are presented in Table 11.

**Table 11:** Heterogeneous effects.

	1. Hukou	2. Gender
	(1)	(2)
Parental income	0.167**	0.197***
	(0.067)	(0.060)
CEL	28.764**	19.743
	(11.783)	(13.846)
Parental income × CEL × Non-agricultural hukou dummy	−0.494**	
	(0.227)	
Parental income × CEL × Male dummy		0.210
		(0.288)
Inverse Mills ratio	4.563	4.388
	(4.080)	(4.289)
Observations	2,258	2,258
R^2^	0.335	0.328

Robust standard errors clustered by province in parentheses. ***p < 0.01, **p < 0.05, *p < 0.1. Estimates are based on Equation 4. Control: CEL, parental income, male (Column 1), non-agricultural hukou (Column 2), age, age^2^, birth cohort, province, province trends, and all two-way interactions between parental income, CEL, and the heterogeneity variable.

#### 5.5.1 Hukou

Column 1 of Table 11 reports the estimation results for hukou heterogeneity. The coefficient of the three-way interaction term is negative and significant, indicating that the CEL had a stronger effect on intergenerational income mobility among individuals with non-agricultural hukou. Given that the CEL generally reduced the intergenerational income correlation, a negative coefficient implies a larger decline in the parental income gradient for urban children, suggesting stronger mobility gains among urban families. Although this finding contrasts with the expectation in Hypothesis 5, it can be explained by policy implementation and mechanism heterogeneity. The stronger implementation intensity of the CEL in urban areas may have led to greater reductions in junior secondary inequality, while in rural regions the law may have increased inequality in senior secondary education and high-status occupational opportunities, producing stronger offsetting effects. The CEL may have widened the urban–rural gap in intergenerational mobility, thereby constraining progress in reducing economic stratification.

#### 5.5.2 Gender

Column 2 of Table 11 reports the estimation results for gender heterogeneity. The coefficient of the three-way interaction term (Parental income × CEL × Male) is positive but not statistically significant, suggesting that the CEL’s effect on intergenerational income mobility does not differ significantly between men and women. This finding does not align with our expectations and may be explained by differences in the underlying mechanisms across genders. For example, the CEL may have reduced inequality in access to non-agricultural employment opportunities more substantially for men. Given the mixed evidence in the literature (e.g., Chen et al., 2021, who find larger gains for men), future research should further examine potential gender-specific effects.

## 6 Conclusion

This study draws on data from the China Household Income Project and applies a difference-in-differences design to estimate the causal effect of the 1986 Compulsory Education Law (CEL) on intergenerational income mobility, while addressing income measurement errors and potential sample selection bias due to co-residence. The results demonstrate that CEL significantly enhanced intergenerational income mobility, and the results remain robust across a series of robustness and sensitivity checks. Mechanism analysis indicates that the policy significantly reduced inequalities in access to junior secondary education and non-agricultural employment opportunities, thereby enhancing intergenerational income mobility. In contrast, inequalities in access to senior secondary education, high-status occupations, and class-based differences in returns to education partially offset these gains. Heterogeneity analysis further reveals that the CEL exerted a stronger impact among individuals with urban (non-agricultural) hukou, whereas no significant gender differences were observed.

These findings suggest that CEL reduced the dependence of children’s income on family background by equalizing access to education through institutional reform. The decline in inequality in access to junior secondary education and the improvement in equality of non-agricultural employment opportunities created crucial channels of social mobility for low-income families, contributing to narrowing income disparities and promoting social equity. In contrast, the increase in inequalities in access to senior secondary education and high-status occupations suggests that advantaged families continued to maintain their educational advantages through family resources, which translated into occupational advantages for their children. This process further widened class-based disparities in income and partially constrained social mobility. The predominance of the mitigating mechanisms can be attributed to two main factors. First, although the heterogeneity analysis indicates that the CEL had a stronger effect among urban families, the policy reached a much broader population and directly improved educational access for rural and low-income households previously constrained in obtaining compulsory education. Second, from the 1980s to the late 1990s, the substantial expansion of junior secondary education, reflected in rising enrollment and completion rates, together with the growth of non-agricultural employment, created broader channels for structural mobility that were not entirely dependent on the policy itself. In contrast, the offsetting mechanisms related to inequalities in senior secondary education and high-status occupational attainment affected only a relatively small segment of the population.

This study contributes to research on intergenerational mobility by analyzing educational inequality through the lens of sociological frameworks such as the Maximally Maintained Inequality and Effectively Maintained Inequality models in a developing-country context. The mechanism analysis also draws on the concept of structural mobility to examine occupational opportunity inequality, particularly access to non-agricultural employment, distinguishing these structural factors from the intergenerational transmission of high-status occupations. Since intergenerational income mobility arises from the combined effects of educational attainment and labor market structures, this integrated perspective offers a more comprehensive understanding than analyses of educational or occupational mobility alone. The findings demonstrate that institutional equalization in compulsory education can counterbalance class-based advantages accumulated through upper-secondary schooling and access to high-status occupations, providing nuanced insights into how educational reforms interact with social structures to shape long-term income mobility in transitional economies.

Methodologically, this study demonstrates how integrating quasi-experimental and selection-correction approaches can enhance the causal assessment of intergenerational processes. The difference-in-differences design enables credible identification of the CEL’s causal effects, while the Heckman correction addresses potential co-residence selection bias that is often neglected in sociological research on income mobility. To further alleviate income measurement error, the study adopts several standard strategies in empirical economics, such as using income ranks and age controls. Although the inverse Mills ratio is generally insignificant in the baseline and robustness analyses, it becomes significant in the mechanism analysis related to educational opportunity inequality (Table 8). This pattern indicates that potential co-residence selection bias may differ across subsamples and model specifications. Applying a selection model remains valuable even when the inverse Mills ratio is statistically insignificant, as it helps verify the absence of selection bias and reinforces the robustness of the findings. This methodological integration strengthens the robustness of the findings and offers a practical framework for future interdisciplinary research on intergenerational income mobility.

By linking causal identification with sociological theory, this study situates the CEL within a broader comparative framework of social mobility research. It demonstrates how combining sociological perspectives with econometric approaches can deepen our understanding of how institutional reforms reshape opportunity structures in transitional economies. Beyond the Chinese context, these findings invite further comparative inquiry into how policy-driven educational equalization affects patterns of social stratification and intergenerational mobility across developing societies.

From a policy perspective, the study underscores the importance of optimizing resource allocation in the compulsory education system, especially in rural and underdeveloped regions. Enhancing educational quality and consolidating the progress achieved in equalizing access to junior secondary education can further reduce the influence of family background on educational opportunities. As inequalities in access persist at the senior secondary level, policies should enhance educational equity by improving access to high-quality schools and ensuring that children from rural and low-income families gain equitable access to academic advancement, thereby promoting broader social mobility and income equity. The findings also provide empirical support for extending the duration of compulsory education as a potentially effective policy measure to enhance intergenerational mobility and advance social equity. More broadly, the study provides empirical insights into social mobility in developing economies, particularly regarding how educational policies can reduce class stratification and foster social inclusion.

Due to data limitations, the current analysis is restricted to a relatively short observation period. Future research could draw on longer-term data to investigate the dynamic effects of the Compulsory Education Law. In addition, focusing on its role in promoting upward intergenerational income mobility represents a promising direction for further inquiry.

## Data Availability

Publicly available datasets were analyzed in this study. This data can be found here: http://chip.bnu.edu.cn.

## Footnotes

^1^The data are obtained from multiple years of the *China Statistical Yearbook* and provincial statistical yearbooks. Information on primary school graduation transition rates is not available for Hainan, Sichuan, and Chongqing.

^2^Pekkarinen et al. (2009) employed this approach to examine the effect of comprehensive school reform on intergenerational income mobility in Finland. By contrast, some studies on the CEL and higher education expansion omitted the interaction terms $\varphi Y_{p}*D_{j}$ and $\pi Y_{p}*D_{t}$. For comparison, this paper also reports estimation results based on this simplified specification.

^3^Tibet implemented the CEL in 1994, but it is not covered by the CHIP survey sampling frame; therefore, no data are reported in Table 2.

^4^Several studies restrict samples to critical age ranges affected by the education policy, such as individuals aged 10 to 20 at the time of implementation (i.e., within three to 5 years of the cutoff age). Consistent with this approach, we conduct robustness checks using a similar sample selection criterion.

^5^Due to data limitations, treatment status is assigned based on birth year rather than the exact birth month. Individuals born in the cutoff year are treated as fully exposed, since the CHIP 1995 and 2002 waves report only completed age. Robustness checks exclude cutoff-year births to address potential misclassification.

^6^Some studies define treatment as 1 only for those under age 6 at implementation and 0 otherwise (e.g., Huang, 2015). This is problematic because (a) children aged 6–15 were still partially exposed, and (b) such a coarse cutoff may confound the effects with contemporaneous policies such as the One-Child Policy and the 1999 higher education expansion.

^7^Because of parent–child co-residence bias, the sample overrepresents males and underrepresents urban and non-agricultural households. We address this issue using a Heckman selection model.

^8^While the results are robust to year-of-birth fixed effects, cohort fixed effects are used for brevity in presentation.
